# Policies and guidelines supporting the sustainability of human milk donation to milk banks in Switzerland: a document analysis

**DOI:** 10.1186/s12939-025-02591-3

**Published:** 2025-07-31

**Authors:** Christelle Kaech, Tracy Humphrey, Catherine Kilgour, Céline Julie Fischer Fumeaux, Claire de Labrusse

**Affiliations:** 1 School of Nursing, Midwifery and Social Work, Faculty of Health and Behavioural Science, The University of Queensland, St Lucia, QLD Australia; 2 HESAV School of Health Sciences - Vaud, HES-SO University of Applied Sciences and Arts Western Switzerland, Lausanne, Switzerland; 3 Clinical Health Sciences, University of South Australia, Adelaide, Australia; 4 The Royal Brisbane and Women’s Hospital, Queensland Health, Herston, QLD Australia; 5 Department Mother-Woman-Child, Clinic of Neonatology, Lausanne University Hospital, Lausanne, Switzerland; 6 Faculty of Biology and Medicine, University of Lausanne, Lausanne, Switzerland

**Keywords:** Milk banking, Donation sustainability, Systematic qualitative review, Document analysis, Guidelines and policies

## Abstract

**Background:**

Donor milk from a human milk bank is recommended for vulnerable infants when the mother’s own milk is scarce. Globally, despite an increasing number of human milk banks, the demand for donor milk exceeds the supply, and access remains inequitable and insufficient. The objective of this document analysis was to investigate how global (universal), European, and national policies and guidelines support the sustainability of human milk donation in Switzerland.

**Methods:**

Database searches of Medline (via PubMed) and CINAHL were completed in June 2024. Eight documents were included: three guidelines, two position papers/consensus statements, two toolkits and one document with policy recommendations. The analysis was constructed around the micro (individual), meso (institutional) and macro (healthcare system and policies) level framework of structures and systems.

**Results:**

Global and European documents offered general recommendations that can be flexibly adapted for each location. Global documents contained explicit recommendations related to sustainability for donations mainly at the macro and meso levels, whereas European documents recommendations related to factors influencing sustainability of human milk donation at all three levels. Swiss guidelines primarily addressed the meso level through specific recommendations adapted to the national context. Regarding sustainability and its three pillars, the majority of the identified recommendations focused on the social pillar. The economic pillar was moderately addressed, whereas the environmental pillar—encompassing issues such as milk wastage, contamination, pollution associated with single-used plastics and broader environmental impacts—was largely overlooked.

**Conclusions:**

The sustainability of human milk donation is addressed inconsistently in current global, European and national guidelines on human milk banking. Some documents provide multiple explicit recommendations across different levels, while others refer to the sustainability of donation only implicitly. Future work and research should consider providing a coherent framework across policy, organisational and behavioural levels to enhance the sustainability of human milk donation.

**Supplementary Information:**

The online version contains supplementary material available at 10.1186/s12939-025-02591-3.

## Background

Donor human milk (DHM) from a human milk bank is recommended as the best alternative to feed infants in vulnerable situations (e.g. prematurity) when their mothers’ own milk is scarce [1, 2]. In these cases, the World Health Organization (WHO), the European Society for Paediatric Gastroenterology, Hepatology and Nutrition (ESPGHAN), the American Academy of Pediatrics (AAP) and other professional reference expert groups encourage the use of DHM, with priority given to very preterm (< 32 weeks’ gestation) or very low birth weight (< 1.5 kg) infants [3–5]. Notably, among other advantages, the use of DHM rather than infant formula in very preterm infants has been shown to decrease the incidence of necrotising enterocolitis [6].

Human milk banks are responsible for collecting, processing and delivering safe DHM to infants in need, while contributing to protecting, promoting and supporting breastfeeding [7, 8]. They recruit and screen donors, and then collect, process, store and distribute DHM [9, 10]. The demand for DHM is high, as an estimated 13–15 million premature infants (< 37 weeks of gestation) are born every year worldwide [11, 12]. Of those, more than two million (15%) are very preterm (< 32 weeks’ gestation) and have a higher risk of morbi-mortality [13]. In 2020, despite a lack of quality data, only 800,000 infants were estimated to receive DHM every year worldwide, provided by more than 700 milk banks in over 60 countries [7, 14–16].

The locations and organisation of human milk bank accessibility vary across the world, with a higher concentration of milk banks in Europe, Brazil and North America, whereas human milk banks are relatively few in regions such as Asia and Africa [7]. The use of DHM also differs between and within nations according to various factors, such as the existence of supportive policies, awareness about human milk donation, costs of operating milk banks and existing resources to milk banks [7, 17]. The current global demand for DHM exceeds the supply, and important disparities exist across the world.

The need to address this DHM supply problem has been increasingly reported over the past five years [18, 19]. In addition to the development and organisation of human milk banks, a need remains for strategies enhancing the sustainability of DHM to ensure adequate and equitable supply. Indeed, a shortage of DHM due to insufficient milk supply is frequently reported by human milk banks [20] and the situation may worsen locally in times of crisis, as occurred for some milk banks during the COVID-19 pandemic, particularly when pragmatism and clear guidance are lacking and communication between milk banks is limited [14, 16]. For these reasons, the importance of a sustainable DHM supply is highlighted in several documents and warrants the resulting calls for action [18, 21].

Sustainability is considered to be ‘the capability of being maintained at a certain rate or level’ [22]. In this paper, we define ‘sustainability’ as the ongoing availability of DHM, along with the operational and financial viability of DHM banking systems. This framing aligns with the emphasis by PATH Global Implementation Framework on sustaining supply, maintaining quality and supporting equitable access to DHM as a bridge to the mother’s own milk [21]. Sustainability is often viewed as having three pillars or perspectives: (1) social, which considers the population and its well-being; (2) environmental, which includes resources and wastage; and (3) economic, which maximises cost efficiency and cost benefits [23]. In this paper, we examine the sustainability of human milk donation through the lens of these three pillars. The evidence-based literature on the sustainability of human milk donation and human milk banking remains limited. In our recent systematic literature review exploring the factors influencing the sustainability of milk donation to human milk banks, we identified 30 of these factors [24]. Most factors were at the individual (micro) level (*n* = 26 related to donors’ and/or their infants’ features) and some were at the institutional (meso) level (*n* = 4 related to milk banks), but none were at the macro level (healthcare and milk banking systems).

In the present study, we chose to explore the influence of policies and guidelines on human milk donation across all levels, focusing on Switzerland. Switzerland is in the middle of the European continent and has a federal constitution consisting of 26 states and a challenging and complex health system organisation. The country has four official languages: German, French, Italian and Romansh. The birth rate in Switzerland is around 80,000 births per year. Approximately 6–7% of those are premature and approximately 1% are very premature births [25]. Switzerland currently has nine human milk banks, with an uneven distribution across different geographic and linguistic regions [26]. All milk banks are attached to hospitals and differ in their organisations, sizes, practices and financial support [27].

As with most European countries, Switzerland currently has no legal or regulatory statutes for human milk and human milk banks [28]. Nevertheless, national guidelines have been produced and revised over the past decade to guide, reinforce and develop Swiss human milk banks’ organisation [9, 29].

The contributions that policies and guidelines make to the sustainability of human milk donation at micro/meso levels and their broader impact remain unclear. This review investigates how global (universal), European and national policies and guidelines support the sustainability of human milk donation to human milk banks in Switzerland at the macro, meso and micro levels. In this paper, a policy is defined as ‘a principle or a course of action adopted by a government’, while a guideline is ‘a set of rules or a general statement to guide a process or procedure’ [30, 31].

## Methods

This qualitative systematic review was performed using a document analysis approach inspired and adapted from the Altheide and Schneider (2013) model which has 12 steps organised into five stages [32]. The study protocol and details of the literature search strategy are available on the international prospective register of systematic reviews (PROSPERO) (#CRD42023395899).

### Search strategy and selection criteria

The literature search strategy was developed by the first author and reviewed by the co-authors and by librarians from both affiliated universities. The literature was searched in January 2023 (with an updated search conducted in June 2024) using Medline (PubMed) and Cumulative Index to Nursing and Allied Health Literature (CINAHL) databases. Manual searches were performed on Google, Google Scholar and the websites of various professional associations, organisations and other entities such as the European Foundation for the Care of Newborn Infants (EFCNI), the European Milk Banking Association (EMBA), the European Directorate for the Quality of Medicines & Healthcare (EDQM) and PATH, among others. The lists of references in the guidelines found during the literature search were also searched to identify potential additional guidelines (snowballing references).

The search terms included ‘milk bank*’, ‘human milk’, ‘donation’, ‘donor*’, ‘sustainability’, ‘policies’, ‘guideline*’ and ‘recommendation*’ (see Table 1). The equations for each database, the filters/limits and the dates of the last search are available in Supplementary material Tables S1a and S1b.

**Table 1:** Literature retrieval strategy in pubmed

Number	Specific search content
#1	human milk OR milk
#2	donor OR donors OR donat* OR milk donation* OR donor human milk OR human donor milk OR human milk donor*
#3	Guidelines as Topic OR Practice guidelines as Topic OR Guideline OR Health Planning Guidelines OR Practice Guideline OR Standard of Care OR Evidence-Based Practice OR Evidence-Based Medicine OR Policy OR Organizational Policy OR Public Policy OR Policy Making OR Health Policy OR Guideline* OR recommendation* OR Principle OR Rule* OR regulation* OR polic* OR advice* OR Code* OR “proposed action* OR system* OR Procedure* OR politic*
#4	milk banks OR milk bank OR milkbank
#5	((#1 AND #2) OR #4) AND #3

The term ‘sustainability’ was found to restrict the search and to hinder our ability to locate relevant documents; therefore, it was removed from our search strategy. This was primarily because, although sustainability-related considerations were explicitly or implicitly addressed within these documents, they were not reflected in the titles or abstracts. To overcome this challenge, objective sustainability-related criteria were systematically applied through a structured data extraction grid, which was uniformly implemented across all selected documents.

To ensure that all local and national policies were identified, Fedlex (a Swiss-legislated publication platform) was also searched, as were the official websites of the Cantons (states) in which a Swiss milk bank is located (see the list in Supplementary Table S2). All nine Swiss human milk banks were contacted by email and asked to share their local guidelines or protocols about milk banking governance (targeting health care professionals, not donors). Five of the nine human milk banks responded. Four sent documents targeting donors (not milk bank staff or policy) that were outside of the scope of this review (e.g. information sheets about milk banks and human milk donation, protocols for donors regarding hygiene and pumping, or donor screening documents).

The inclusion criteria were current global, European or Swiss national policies and guidelines or recommendations that were focused on supporting (explicitly or implicitly) the sustainability of human milk donation. In this study, the term ‘European’ refers to both pan-European documents and multi-country European documents that include Switzerland. Only policies and guidelines (including documents self-defined as position papers and toolkits) relevant to Switzerland—meaning they either target users in Switzerland or pertain to the country geographically (e.g. national, European or global)—were included. All available documents were considered (no date limits). When multiple versions existed, only the most contemporary version was included. Exclusion criteria were as follow: (1) guidelines focusing mainly on the processing or use of DHM and (2) documents not written in English or one of the languages of the Swiss milk banks (French and German). Documents were excluded if they were not relevant or applicable to the case context (see the Discussion for further details) [33, 34].

**Fig. 1:**
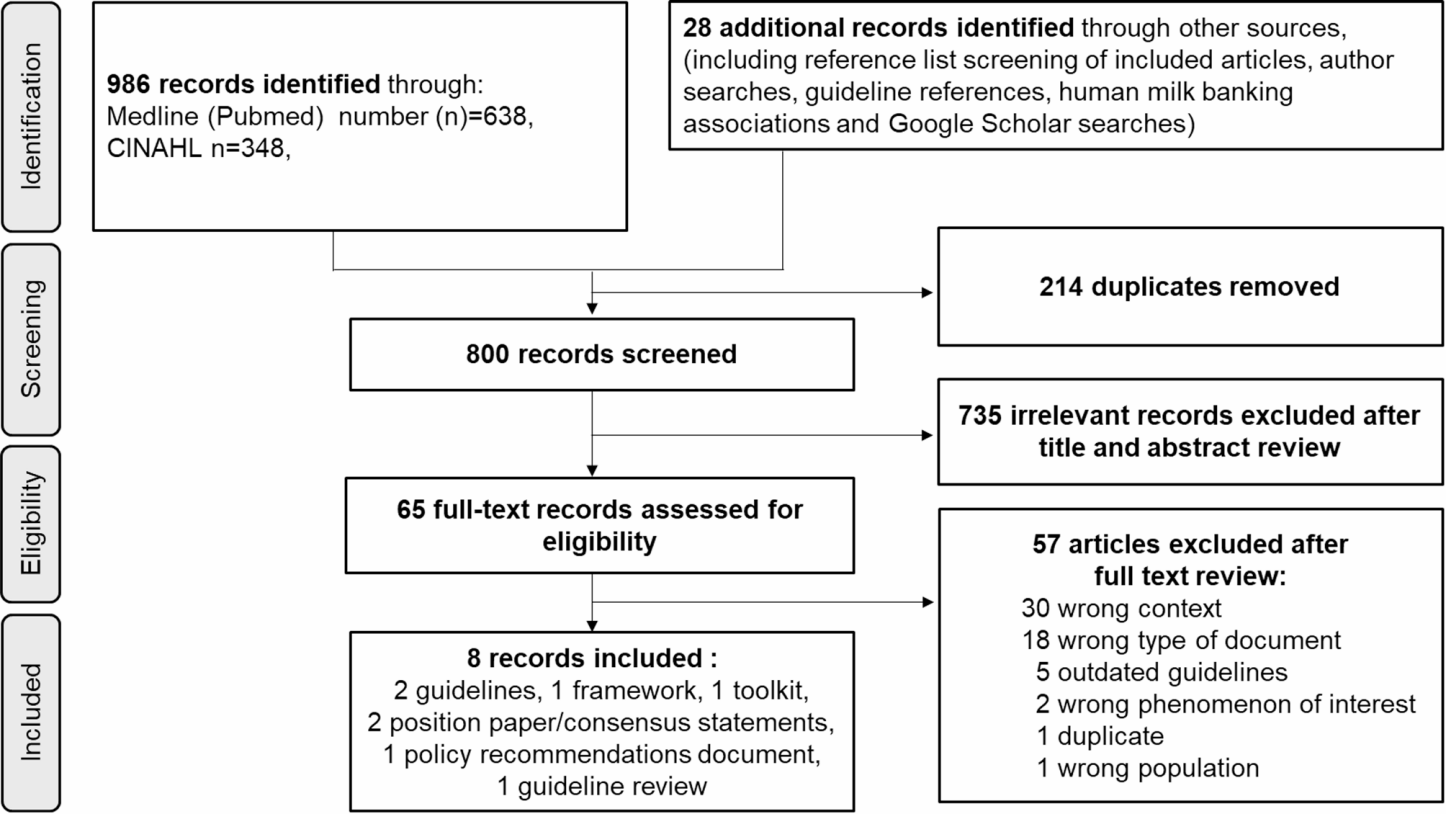
PRISMA flow diagram of the study selection

All the documents found were uploaded into COVIDENCE and duplicates were automatically removed. The first and one of the last co-authors of this article screened all the documents against the inclusion and exclusion criteria. Discrepancies were discussed, and other co-authors were involved when needed. The selection process is detailed in the PRISMA flow diagram (Fig. 1) and in the Results section.

### Data extraction and analysis

The first author developed the extraction grid, based on existing tools and literature, to include document information, considerations related to the sustainability of milk donation (including its three pillars) and existing recommendations [24, 32, 35–38]. Following peer review by one of the last authors, this extraction grid was used to collect data. A list of all the items included in the grid is presented in Supplementary Table S3. Refinement of the grid continued during data extraction. The first author assessed the quality of the documents using the validated AGREE II tool [39]. The data extraction and document quality were checked by a co-author for two of the documents (> 25%) to ensure the quality and validity of the process. Differences between the two authors were discussed until a consensus was reached.

Following extraction, the key information was organised by themes. The process of developing the themes relied on both deductive and inductive methods. Hence, the findings were organised in three ways. The first consisted of classifying the documents according to their relevance and setting (e.g. global, European or Swiss national documents). Second, the findings (factors and recommendations supporting the sustainability of human milk donation) were categorised into three different levels as macro (e.g. healthcare systems, human milk bank systems, policies), meso (related to institutions such as milk banks or hospitals) or micro (linked to individuals such as donors) factors. Third, we reported how each level engages with sustainability in relation to the three pillars of sustainability (social, economic, and environmental). This analysis highlighted the differences and similarities across the themes and documents and enabled the narrative synthesis of the documents.

The preferred reporting items for systematic reviews and meta-analyses (PRISMA) tool was used to structure and report this qualitative document analysis informed by a systematic search step [40]. The completed PRISMA checklists are available in Supplementary Tables S4 & S5.

### Patient and public involvement

No patients or members of the public were involved in the design, conduct, reporting or dissemination of our research.

## Results

In total, 1014 records were identified, with eight meeting the eligibility criteria (Fig. 1) [9, 10, 21, 41–45]. This search yielded guidelines, frameworks, toolkits, policy recommendations, but no actual policies on human milk banking.

### Characteristics of the included documents

All eight included documents were published between 2017 and 2022. Seven were in English and one was in German. Two were guidelines, two were frameworks and/or toolkits, two were position papers/consensus statements, one highlighted policy recommendations and one was a review of PATH guideline (Table 2). Of these eight documents, two were global and five were European (two of which targeted German-speaking European countries more specifically). The last document included was a national Swiss guideline. The main results are displayed in Tables 3 and 4.

**Table 2:** Characteristics of the eight included documents (from global to national, then alphabetically by authors)

Authors/ organisation	Type of document	Edition/ pages number	Title	Target audience /users	Reference to other included documents	
GLOBAL GUIDELINES/DOCUMENTS						
DeMarchis et al. (2017) [10]	Guidelines	1st Edition / 6 pages	Establishing an Integrated Human Milk Banking Approach to Strengthen Newborn Care	Policy makers, health leaders who are developing HMB* strategies, existing HMB systems	PATH (2013)	
PATH* (2019) [21]	Global Implement-ation Framework (& toolkit)	2nd Edition / 82 pages	Strengthening Human Milk Banking: A resource Toolkit for Establishing & Integrating Human Milk Bank Programs. A global Implementation Framework. Version 2.0	Ministries of health, policymakers, implementers, existing and emerging HMBs*. Global human milk and breastfeeding community. Health departments, healthcare facilities and health workers.	EDQM (2017), Frischknecht et al. (2010)**[29], DeMarchis et al. (2017)	
EUROPEAN GUIDELINES/DOCUMENTS						
EDQM* (2022) [41]	Guidelines	5th Edition / 704 pages	Guide to the Quality and Safety of Tissues and Cells for Human Application (5th Edition)	Professionals involved in donation, banking, transplantation and other clinical applications of tissues and cells	PATH (2013), Weaver et al. (2019)	
EFCNI* (2018) [42]	Position paper	1st Edition / 28 pages	Recommendations for Promoting Human Milk Banks in Germany, Austria, and Switzerland.	HMBs and hospitals interested in establishing a human milk bank in Germany, Austria or Switzerland	Frischknecht et al. (2010)	
EFCNI* (2018) [43]	Toolkit	1st Edition / 44 pages	Toolkit for Establishing and Organising Human Milk Banks	HMBs and hospitals interested in establishing a HMB in Germany, Austria or Switzerland	EFCNI (2018) [42] Frischknecht et al. (2010)**	
EFCNI* (2020) [44]	Policy recommendations	1st Edition / 28 pages	Making Human Milk Matter. The Need for Regulation in the European Union. Policy Recommendations	European policy makers / legislators.	Weaver et al. (2019), EFCNI (2018) [42], EDQM (2019),	
Weaver et al. (2019) [45] EMBA*	Consensus statement	1st Edition / 8 pages	Recommendations for the Establishment and Operation of Human Milk Banks in Europe: A Consensus Statement from the European Milk Bank Association	European milk banks and European countries that do not have HMBs.	PATH (2013)	
NATIONAL SWISS GUIDELINE/DOCUMENT						
Ahrens et al. (2020) [9]	Guidelines	2nd Edition	Leitlinie zur Organisation und Arbeitsweise einer Frauenmilchbank in der Schweiz [Guideline on the Organisation and Operation of a Human Milk Bank in Switzerland].	Swiss HMBs and their staff	PATH 2013, Frischknecht et al. (2010), EDQM (2017)	

*EFCNI = European Foundation for the Care of Newborn Infants, EDQM = European Directorate for the Quality of Medicines & Healthcare, EMBA = European Milk Banking Association, HMB(s) = Human milk bank(s) **first version of the Swiss guidelines

**Table 3:** Themes of sustainability of human milk donation, organised by document and levels

Authors/ organisation	Sustainability DHM donation		Micro level (donors)	Meso level (milk banks)	Macro level (systems)	Added value of the document
GLOBAL GUIDELINES/DOCUMENTS						
DeMarchis et al. (2017) [10]	Explicitly discussed but not defined.		Free-of-charge donation (for donors)	Lactation support. Location of HMB* to facilitate donation. Community engagement and promotion of donation.	Policy support at local, national and international levels. Integration of DHM* provision into infant nutrition, guideline, and care policies. HMB network. HMB system integration with hospital and community.	The document is developed from PATH guideline. It discusses some important aspects of sustainability of donation and sustainability of human milk banking systems.
PATH* (2019)[21]	Explicitly discussed and defined.		Voluntary and uncompensated donation	Timing and duration of donation. Lactation support for mothers. Exclusive breastfeeding protection, support and promotion. Encouragement to donate milk excess. Donors’ recruitment through various channels.	Local, national and global support. National policies.	Comprehensive framework, including the concept of sustainability in human milk banking. It provides universal tools and standards on human milk banking.
EUROPEAN GUIDELINES/DOCUMENTS						
EDQM* (2022) [41]	Explicitly discussed but not defined.		Bereaved mothers. Milk collected prior to becoming a donor.	Promotion of DHM donation through various channels; new donor training (hygiene, collecting and expressing milk, among others). Staff training (which includes hygiene, quality control, safety and traceability among others). Public awareness strategies and donor recruitment.	Human milk legal status, regulation at national and European level, guidelines development).	Most of the recommendations are specific. The document recommends promoting and supporting human milk donation by having adequate numbers of trained-staff members who are prepared and motivated. The document cites ways to promote human milk donation.
EFCNI* (2018) [42]	Implicitly addressed through considerations related to sustainability		Mothers of preterm and ill infants who have an excess of milk are allowed to donate. Donors’ infants were often previous recipients of DHM.	Establish more HMBs. Increase number of donors to meet the demand. Share resources between centeres, better repartition of resources. Support donors and strengthen the relationship with donors.	Public and financial support from the health system essential.	The purpose of this position paper is to promote human milk banks in Germany, Austria and Switzerland. This could indirectly facilitate the donation to milk banks
EFCNI* (2018) [43]	Implicitly addressed through considerations related to sustainability		Recruit mothers of preterm or newborn infants. Take into consideration: donor’s milk supply, child’s gestational age and mother’s hygiene standards, linguistic communication and religious background.	Donor recruitment channels. Information about human milk donation: materials and channels.	Legal regulation	The document offers examples of information materials for donors, information material on the donation process for health care professionals and checklists for explanatory meetings on breastmilk donation, as well as a data sheet on pumping, collecting and transportation of donor milk.
EFCNI* (2020) [44]	Implicitly addressed through considerations related to sustainability		X	X	Regulatory framework	This document is a call for regulation in the European Union. It indirectly supports the donation of milk to HMB at a macro level.
Weaver et al. (2019) [45] EMBA*	Explicitly discussed, but not in relation to supply or donation		Bereaved mothers and personal hygiene practices related to milk expression	Infrastructure, staffing, donor milk safety and quality, donor screening	X	First Europe-wide consensus statement (from the EMBA). This could indirectly facilitate donations to milk banks.
NATIONAL SWISS GUIDELINE/DOCUMENT						
Ahrens et al. (2020) [9]	Explicitly discussed regarding economic and legal considerations of HMBs, but not of supply or donation.		Donors and their infants’ characteristics (but does not link them to sustainability of donation and supply)	Recommends limiting human milk donation to 6 months postpartum and offering information and instruction on breast pump use, hygiene and cleaning steps to minimise wastage due to bacterial contamination.	Some important elements were included that were not explicitly linked to sustainability of donation in the guideline, but were considered related in the global documents.	The recommendations are detailed and specific to the Swiss context and are well referenced. The need for a legal framework for DHM and general health policy and financing models to support HMBs in Switzerland was highlighted in the guidelines.

*EFCNI = European Foundation for the Care of Newborn Infants, EDQM = European Directorate for the Quality of Medicines & Healthcare, EMBA = European Milk Banking Association, DHM = Donor Human Milk, HBMs = Human milk banks **First version of the Swiss guidelines

**Table 4:** Considerations related to social, economic and environmental pillars of sustainability at each level*

Level	Sustainability pillars	Global documents			European documents								Swiss national document
		DeMarchis et al. (2017) [10]	PATH (2019)[21]		EDQM** (2022) [41]		EFCNI** (2018) [42]		EFCNI** (2018) [43]	EFCNI** (2020) [44]		Weaver et al. (2019) [45] EMBA**	Ahrens et al. (2020) [9]
Macro	Social	Policies	Policies & regulation		Regulatory framework		Establishment of further HMBs		Legal regulation	Regulatory framework		X	Regulation at national level.
	Economic	Financial support	HMBs funding, financial resources, cost		Funding and resources		Regulated financial framework		X	X		X	X
	Environmental	X	X		X		X		X	X		X	X
Meso	Social	Promotion and support of BF, staffing skills and training	Recruitment channels, lactation support and education, equipment provision for donors, donor screening, staff training		Recruitment channels, training (staff, donors), donor screening, donor milk safety & quality.		Donors’ support, donor screening, infrastructure, staffing, donor milk safety and quality.		Recruitment channels, staff training, donor milk safety and quality, infrastructure and staff, Donor screening.	X		Infrastructure, staffing, donor milk safety and quality, donor screening	Infrastructure, staffing, donor milk safety and quality, donor screening
	Economic	Costs of an HMB	Operational costs of an HMB		X		Resource sharing between HMBs		Operational costs of an HMB	X		X	X
	Environmental	X	Screening for potential environmental contaminants		X		X		X	X		X	Potential environmental contaminants and wastage
Micro	Social	Target donors with milk in excess of their own infants’ needs	Target donors with milk in excess of their own infants’ needs		Milk excess, legal age, bereaved mothers		Donor characteristics (mothers of preterm & ill infants)		Donor characteristics (mothers of preterm, milk supply, infant gestational age). Personal hygiene practices related to milk expression	Donor characteristic (breastfeeding women with milk in excess)		Health behaviour and status. Donor characteristics (bereaved mothers), Personal hygiene practices related to milk expression	Donors and their infants’ characteristics and extent of milk excess, personal hygiene practices related to milk expression
	Economic	Donation free-of-charge for donor.	Donation without payment, but reimbursement to the donor for incurred costs mentioned		Donation unpaid		Donation not compensated		Donation unpaid	X		X	Free of charge for donors. Donors receive no financial compensation.
	Environmental	X	X		X		X		X	X	X		X

*Not an extensive list, but main examples of the considerations covered in the documents

**EFCNI = European Foundation for the Care of Newborn Infants, EDQM = European Directorate for the Quality of Medicines & Healthcare, EMBA = European Milk Banking Association, DHM = Donor Human Milk, HBMs = Human milk banks**first version of the Swiss guidelines

The quality of each document, as assessed using the AGREE II tool, varied from fair to adequate [39]. This score reflects the fact that most of the appraised documents were not guidelines. Rather, they were toolkits, consensuses or position papers, which do not always involve users (donors or recipient families, for instance) and often lack transparency regarding document development, methods or quality of the evidence.

All eight documents clearly stated that DHM should be offered to human milk banks voluntarily and freely, and five documents explicitly stipulated that donations should not involve financial incentives [9, 10, 21, 41–45]. Providing financial incentives to donors was viewed as potentially jeopardising their health or the health of their infants, thereby raising ethical and safety concerns [21].

### Synthesis of the findings of global documents

Two global documents were found; one is a framework/toolkit [21] and one is a guideline [10]. Both documents are closely related. Both highlight and discuss the importance of the sustainability of the DHM supply and of a sustainable human milk bank system. The recommendations focus on providing essential resources to incorporate human milk banking into breastfeeding support and neonatal care. The documents contain recommendations that are appropriately generalisable and can be adapted to specific contexts (e.g. country, environment, setting). They are aimed at policy makers, health leaders, human milk banks and health care professionals, among others.

At the macro level (policies, health and human milk bank systems), these two global documents emphasise various recommendations regarding factors that explicitly or possibly influence the sustainability of human milk donation [10, 21]. The authors highlight that regulation, policies, and guidance are needed at the global, national and local levels to support a sustainable supply of DHM. A strong integration of human milk bank systems across hospitals and communities is recommended, with an emphasis on the importance of collaboration and networking among human milk banks, as well as the protection, promotion, and support of breastfeeding, all of which are implicitly linked to sustainability. Regarding the pillars of sustainability, the documents address elements pertaining to the social and economic pillars but do not cover environmental aspects at the macro level (see Table 4 for further details).

At the meso level (milk banks and healthcare providers), the recommendations relate to lactation support, donor recruitment and support of donors, as well as human milk bank locations and facilities and they explicitly relate to sustainability. The documents stress the importance of promoting and protecting exclusive breastfeeding to raise awareness and allow for a larger donor pool. Lactation education and support are proposed to help minimise barriers to donation and to support mothers in building their milk supplies. Once supply is established, mothers should be encouraged to donate the excess milk their own infant does not need.

Several recommendations were made regarding human milk donor recruitment, including communication methods and channels, such as written documents, referrals (e.g. during routine care, antenatal classes, support groups), mass media and the donors themselves (talking with families, peer support groups, and the community). The engagement of human milk banks, local leaders and the community can facilitate the recruitment of donors. Another recommendation was to minimise the burden of the screening process for donors. Regarding the human milk bank location, facilities (space) and the services offered, several recommendations explicitly linked to the idea of a sustainable supply of DHM were made to render the donation process as convenient as possible. For instance, the location of the human milk bank (its accessibility) and a human milk transportation system can have an impact on convenience for human milk donors. Spatial resources, such as lactation support rooms and/or private rooms for breastfeeding and milk expression, were recommended for donors’ comfort. Other elements, such as supporting donors and providing milk collection supplies, are identified. To ensure ongoing quality, training of healthcare professionals and updating of knowledge is recommended. Most of the recommendations at the meso level were focused on the social pillar, whereas some recommendations concerned the economic pillar but only one document mentioned one element pertaining to environmental aspects (see Table 4).

Notably, at the micro level, few recommendations related to the sustainability of donation were discussed implicitly—for example, suggesting that donors with milk in excess of their own infant’s needs should be targeted. Regarding the three pillars of sustainability, aside from the recommendation to target bereaved mothers or donors who have a surplus of milk beyond their own infants’ needs, no specific characteristics of human milk donors were discussed at the social level. Regarding the economic pillar, both documents stated that donation should be free of charge for donors. Environmental aspects were not addressed.

### Synthesis of the findings from European documents

Five European documents were found (one guideline, two position papers/consensus, one toolkit and one policy recommendation) that included factors at all three levels (see following paragraph). The notion of sustainability is explicitly mentioned in one document in relation to the donor milk supply [41], and in another with regard to the overall sustainability of human milk banks [45]. Considerations implicitly related to sustainability and its pillars are present in the remaining three documents [42–44]. The recommendations contained in these documents are appropriately generalisable and can be tailored to specific countries and contexts. This includes the importance of offering good support to donors without limiting these to a particular setting. The identified target audience of these documents is European and includes human milk banks, hospitals, countries that do not have formal human milk banks, healthcare professionals involved in human milk banking, policy makers and legislators.

At the macro level, elements related to sustainability were found but required detailed searching of the document. These documents report that a sustainable supply of DHM requires public and financial support from the national healthcare system. One document also identifies a need for legislation and regulation of human milk and human milk banks, while recommending further research on human milk donation. One of the five documents did not explicitly mention any element or recommendation related to the sustainability of donation at the macro level [45]. The social and economic pillars were addressed in some of the documents, whereas the environmental pillar was largely overlooked.

At the meso level, the following factors were raised: (i) the importance of raising public awareness about donation and education at the local and national levels to facilitate donor recruitment through an adequate public-awareness strategy, (ii) effective recruitment systems (using various channels), and (iii) training for healthcare professionals who recruit donors [41, 43]. Another recommendation was to recruit more donors through public campaigns focusing on altruism and solidarity, among others, to increase the size and diversity of the donor pool. Other elements, such as human milk donation promotion, donor training and healthcare professional training, were mentioned without explicitly relating them to the sustainability of donation. Some also alluded to supporting donors to strengthen their trust in and relationships with human milk banks. Elements related to the social and economic pillars were explored in some of the documents, whereas the environmental pillar was not addressed.

At the micro level, no elements explicitly associated with the sustainability of donation were related to donors or their infants. Some elements implicitly related to sustainability were mentioned, such as the fact that donors should have an excess of milk, that most donors’ infants received DHM while being hospitalised in the neonatal intensive care unit and that human milk donors may donate as an altruistic gesture following their own infants’ use of DHM. Finally, most of the documents point out that the following factors can be considered when recruiting donors: mothers whose children are premature, those with surplus breast milk and bereaved mothers. These elements were not further elaborated on, and their impact on sustainability was not explicitly discussed. Among the five documents, four included recommendations related to the social pillar of sustainability and two addressed considerations related to the economic pillar. In contrast, the environmental pillar was not discussed at the micro level in any of these documents.

### Synthesis of the findings of Swiss national documents

At the Swiss national level, only one national guideline was found that serves as an operational guide for human milk banks and their staff. The guideline does not explicitly address the sustainability of human milk donation but contains considerations that are related to it. The recommendations in this document are rather specific and are oriented to the Swiss context.

At the macro level, although no factors are explicitly related to the sustainability of milk donation or supply, the guideline discusses important elements, such as the need for a legal framework for DHM, general health policy and financing models to support human milk banks in Switzerland. This is considered to pertain to the social pillar; however, the economic and environmental pillars are not addressed at the macro level.

At the meso level, the Swiss guideline recommends limiting human milk donation to six months postpartum. It also advises providing information and instruction on the use of a breast pump, hygiene and cleaning steps to minimise wastage due to bacterial contamination—although none of these measures are explicitly linked to sustainability in the document. These considerations relate to the social and environmental pillars of sustainability, whereas the economic pillar is not addressed at the meso level.

At the micro level, the guideline mentions some elements that were cited in the global and European documents, but without explicitly linking them to the sustainability of donation and supply. For example, the guideline recommends taking the infant gestational age into account when recruiting donors, as well as the volume of milk excess the donors have. The document also recommends that the donation process be free of charge for donors. These considerations relate to social and economic factors, whereas the environmental pillar is not addressed.

Among the included documents that focus entirely on human milk banking, the Swiss guideline, at 222 pages, is the most extensive document. While the EDQM document is longer overall (704 pages), only 11 pages are dedicated to human milk. The Swiss guideline recommendations are tailored for the Swiss context and are well referenced. A synthesis of all the main findings is detailed in Table 5.

**Table 5:** Synthesis of the findings, organised by global, European and Swiss levels

	Findings
Global documents [10, 21] *n* = 2	1. Notion of sustainability explicitly discussed in both documents [10, 21], and defined in one [21].
	2. Recommendations contained in these documents are broad, so that they can be adapted to a variety of specific contexts and countries [10, 21].
	3. Documents include recommendations on factors related to the sustainability of human milk donation at the macro and meso levels, with limited considerations at the micro level [10, 21]. 4. Sustainability considerations were primarily related to the social and economic pillars, while environmental pillar was largely absent [10, 21].
	5. No policies or regulation found.
European documents [41–45] *n* = 5	1. Notion of sustainability is explicitly included, but not defined, in relation to DHM supply in one document [41], and to milk banks in another [45], and considerations implicitly related to sustainability are present in the remaining three documents[42–44].
	2. Recommendations contained in these documents are general, so that they can be adapted to a variety of specific contexts and countries [41–45].
	3. Documents mentioned recommendations on factors that influence the sustainability of human milk donation at the macro, meso and micro levels [41–45]. 4. Sustainability considerations were primarily related to the social and economic pillars, while environmental pillar was not addressed [10, 21, 41–45].
	5. No policies or regulation on human milk and milk banking found.
Swiss National document [9] *n* = 1	1. Notion of sustainability not defined but used explicitly once in relation to economic consideration, and consideration implicitly related to human milk donation sustainability or supply are present [9].
	2. Recommendations contained in this document are detailed and adapted to the Swiss context [9].
	3. Documents includes recommendations on factors related to the sustainability of human milk donation mainly at the meso level, with very few addressing the micro or macro levels [9]. 4. Sustainability considerations were primarily related to the social pillar, with few addressing the economic and environmental pillars [9].
	5. Lack of policies and regulation on human milk and milk banking. 6. No documents or protocols found at the local level for healthcare professionals (most documents identified target donors or are lists of eligibility criteria for donation). 7. Little information is provided on how to support donors or raise awareness about milk donation and milk banks [9]. 8. Little information is provided on how to facilitate an optimal repartition of milk provision and other resources at the national level [9]. 9. Policies and regulation are needed in Switzerland regarding human milk and human milk banking [9].

## Discussion

### Summary of key findings

This qualitative systematic review uses a document analysis process and is, to our knowledge, the first to rigorously examine policies and guidelines for sustaining human milk donations. This review highlights the absence of systematic contemporary policies addressing this issue, revealing that more than half of the documents found do not explicitly discuss the sustainability of donation (or even the sustainability of human milk banking) while containing recommendations that address it implicitly.

### Variation across document types, (micro-meso-macro) levels and sustainability pillars

Variation is found in recommendations according to the document type (e.g. guidelines, toolkit) and the setting (e.g. global, European). Similarities were identified between the global, European and Swiss documents (guidelines, consensus statements and toolkits among others) regarding the general topics covered, such as donor screening, DHM screening and treatment, among others. However, variability was evident in terms of format, length (from six to 704 pages) and comprehension. The scope of the documents, in terms of both their methodology and the precision of their content and recommendations, varies greatly according to the type of documents and its objectives. As intended, the global and European documents state recommendations that are more general, to enable easy adaptation to specific countries, contexts and healthcare systems. Recommendations related to the sustainability of donation were mainly found at the macro and meso levels in the global documents, and at all three levels in the European documents. As can be expected, recommendations that were made in the Swiss guidelines had been adapted to the Swiss context and were directed more to the meso level regarding factors that could relate to sustainability of donation. The recommendations in the Swiss guidelines are rather specific and reflect the diversity of the country’s organisations and funding. This likely reflects the governance of Switzerland and the autonomy of the 26 cantons in determining local health priorities.

### Critical appraisal of quality and transparency

Despite their grades of fair or adequate overall quality, most documents lack transparency regarding their methodology (search methods, databases used, search equations, criteria for selecting the evidence, as well as strengths and limits of the body of evidence). In addition to limited transparency in methodological reporting, disclosure was often insufficient regarding the organisations or experts involved in the development of the selected documents. Some documents included in this review—such as those supported by the Family Larsson-Rosenquist Foundation and the Bill & Melinda Gates Foundation, as well as other sources mentioned in this article such as the ESPGHAN—raised concerns about potential conflicts of interest in relation to the International Code of Marketing of Breast-milk Substitutes, as these organisations have connections to industry stakeholders in infant nutrition [46–48]. Recognising these affiliations is important for understanding the circumstances and mechanisms by which commercial or ideological interests may influence certain recommendations or policy directions.

More than half the documents do not report having included users—such as donors, recipients, and their families—in their development. Including donors can help ensure that their needs and the challenges they face are considered in the co-design of services, ultimately contributing to improved health outcomes. Engaging donors (and their families) also fosters collaborative relationships with human milk banks. PATH, in its global framework, underscores the importance of involving stakeholders—particularly donors—in shaping effective and sustainable human milk banking models [21]. Thus, key recommendations include the systematic inclusion of donors in the development of documents and guidelines and the explicit reporting of this process.

### Concept of sustainability across the included documents

The concept of ‘sustainability’ in the context of donor human milk and human milk banking is not consistently defined across existing documents, and its interpretation varies considerably. While many references emphasise aspects aligned with the social dimension of sustainability and while some address economic considerations, the environmental dimensions are largely neglected. To strengthen global guidance, a need remains for more comprehensive and integrated frameworks that support, protect and promote breastfeeding as well as human milk donation and the development of equitable, sustainable milk banking systems worldwide. An interesting approach might be to integrate these notions more explicitly in the European documents through general recommendations that are adapted to the European context and in the Swiss guidelines through specific and detailed recommendations that are adapted to the Swiss context.

PATH’s recommendations on the sustainability of donations are discussed in depth, and the 10 additional tools provided–such as a guide for developing a communication strategy–serve as practical resources for stakeholders involved in planning and implementing milk banks [21]. Another potential area for improvement is the better integration of human milk banks within the health system, as this could strengthen overall newborn nutrition services and promote equitable access of vulnerable children to an exclusive human milk diet [7]. Better financial and political support, as well as increased collaboration and resources sharing and better repartition of DHM provisions could also help ensure that vulnerable infants have access to DHM.

### Implications for practice

The Swiss guidelines focus on several aspects, such as the infrastructure of milk banks, pathogens in maternal milk, and the selection of donors, but they do not include specific information on how to support donors, nor do they indicate ways to promote and raise awareness about human milk banking. Developing and integrating recommendations on how to facilitate an optimal repartition of milk provision and allocation of other resources (financial, organisational, among others) at the national level would also be an interesting approach, as this could offer greater equity regarding access to DHM and human milk banks. No local documents targeting health care providers (rather than donors) were identified among the five Swiss milk banks that responded (out of nine). One milk bank indicated that the development of its new quality manual, which will include recommendations for health care professionals, is in progress.

Finally, the recommendation to limit human milk donation to six months postpartum is both controversial and inconsistent with other established guidelines. Recommendations vary regarding the duration of milk donation depending on the postpartum age. Although there is a lack of robust scientific evidence on this matter, several existing data do not appear so far to support strong rationales for such limitations [49–51]. This recommendation warrants further scrutiny and investigation, as it may potentially undermine the sustainability of milk donation. The WHO and the United Nations Children’s Fund (UNICEF) recommend exclusive breastfeeding for the first six months, followed by the introduction of safe and nutritionally adequate complementary foods and continued breastfeeding up to two years of age or beyond [52]. Nevertheless, healthy infants who lack sufficient access to their own mother’s milk often face limited alternatives to artificial milk. Access to DHM beyond hospitals is uncommon, and too few guidelines, legislation and research are addressing informal human milk sharing or other alternatives to ensure access to safe and quality human milk for all infants.

On a more global scale, the current development of guidelines for DHM banking by the WHO, will establish minimum standards to ensure the safety and quality of DHM [53]. These guidelines and directives could promote better harmonisation of practices, the quality and safety of milk and, hopefully, the sustainability of milk donations and access to donor milk through milk banks.

### Policy implications

No policies were found during the literature search, and policies are lacking at the global, local or national levels in Switzerland for human milk or human milk banking. The need for national regulations is identified by several authors and organisations [9, 21, 44]. The accompanying lack of legal consideration diminishes the appeal of donation and complicates the operation of milk banks, which would benefit from clear governance. This has created an opportunity for for-profit milk banks to compensate donors, raising ethical dilemmas, such as the risk that donors might prioritise donation over providing maternal milk to their own infants. This could impact the sustainability of donations for non-profit human milk banks, where donors typically do not receive compensation.

One consequence is that non-profit human milk banks may have only limited access to donor milk. This would disproportionately impact resource-restricted families who cannot afford to buy donor milk for-profit milk banks, as well as vulnerable infants in neonatal intensive care units attached to non-profit human milk banks [54]. Five documents included in this analysis discussed issues related to donor compensation, commodification or commercialisation of human milk or human tissues and cells [9, 21, 41–43]. In the literature, some articles discuss the commodification of milk and its implications [54, 55].

Human milk use is not legislated in many countries, including Switzerland, and so it remains unclear how it should be classified (i.e. as food, nutrition therapy, medical product of human origin or body tissue or fluid) [28]. Each classification may have different impacts on DHM cost, accessibility, quality and safety [21]. Notably, in 2024, the European council and the European parliament approved a new regulation to increase the safety and access of substances of human origin [56]. The regulation explicitly includes human milk, and it addresses donor registration as well as the testing and processing of substances of human origin, including human milk. A key goal is to facilitate the cross-border exchange of these substances, within the European Union. Switzerland is not a member of the European Union; however, this might also motivate changes in the country regarding human milk legislation.

### Research implications

A clear need exists for more robust evidence and research on the sustainability of human milk donation, particularly at the macro (e.g. healthcare systems, milk bank systems, national regulations), and meso (e.g. milk bank operations) levels. Documents considered relevant globally and in the European context have often made recommendations on the basis of existing practices in human milk banks, lactation centres and hospitals (to which they might be attached), and rely on national or regional guidelines and/or experts consensus, among others [21, 45]. At the micro level (e.g. donors’ features and their infants’ features), more evidence is also needed to obtain a better understanding of the factors influencing the sustainability of human milk donation in order to strengthen the recommendations [24].

### Limitations and strengths

The rigour of this study is a notable strength, as demonstrated by its comprehensive qualitative document analyses informed by a systematic search step conducted by two researchers using a validated method. To promote the quality of the paper, 25% of the analyses were cross verified by a second member of the research team. To provide details on the quality of the studies, a quality assessment was carried out on all included documents using the AGREE II tool, a high-quality validated tool. This provided detailed information on the assessment of interesting elements of the document’s methodology and quality. Notably, the AGREE II tool has certain limitations, primarily concerning the appraisal of recommendations other than guidelines and the recognition that some of the 23 items are not well adapted to documents other than guidelines.

The goal of this review was not to examine all existing documents from other continents or countries. That type of examination was neither practical nor feasible within the available resources and those documents are likely to be reflected in global and European policies and guidelines; therefore, this was beyond the scope of this review. The documents included in the analysis targeted different settings (with global, European and Swiss focuses); therefore, caution should be exercised when drawing conclusions or making comparisons. This targeting also limits the generalisability and transferability of the findings. However, this allowed us to gain a better grasp of what is documented in the policies, guidelines and other recommendations. It also provides a better understanding of the existing documents that may influence the human milk banking system in Switzerland and the DHM supply, as well as the extent to which they support the sustainability of human donation in Switzerland.

## Conclusions

This is the first known analysis to provide a qualitative document analysis of the extent to which policies and guidelines support the sustainability of human milk donation. It presents these findings within the context of a micro/meso/macro framework and in relation to the three pillars of sustainability (social, economic and environmental).

This document analysis highlights several gaps and areas for improvement. This review joins others in calling for regulation of human milk and human milk banking and highlights the need for breastfeeding and human milk banking strategies at different geographical levels, including the national level in Switzerland. Policies regarding human milk and milk banks are needed to support the sustainability of human milk donation. For example, milk donation could be integrated as a health priority with goals and evaluations, and the status of breast milk could be clarified. In Switzerland, a comprehensive national strategy for breastfeeding and human milk banking is markedly lacking. The implementation of this type of strategy is essential to bolster the availability of human milk for infants and to facilitate the establishment, collaboration and efficiency of milk banks.

Robust research is needed to understand the impact of policies and guidelines on human milk banking and donation and to explore factors influencing human milk banks at the micro (donors and their families), meso (milk banks) and macro (healthcare systems and human milk bank systems) levels. Finally, considerations related to the environmental pillar of sustainability (e.g. wastage, single-use plastics and environmental contaminants) are rarely addressed in the included documents and deserve further exploration and development in research to ensure a better integration of evidence-based recommendations into the available guidelines.

## Electronic supplementary material

Below is the link to the electronic supplementary material.

**Supplementary Material 1**: S1a and S1b search strategy info S2 List of Swiss human milk banks, S3 Extraction grid details, S4 PRISMA, S5 PRISMA Abstracts checklist

## Data Availability

No datasets were generated or analysed during the current study.

## Abbreviations

A: Austria
AAP: American Academy of Pediatrics
CH: Switzerland (Confoederatio Helvetica)
D: Germany (Deutschland)
DHM: Donor Human Milk
EDQM: European Directorate for the Quality of Medicines & Healthcare
EFCNI: European Foundation for the Care of Newborn Infants
EMBA: European Milk Banking Association
ESPGHAN: European Society for Paediatric Gastroenterology, Hepatology and Nutrition
PRISMA: The Preferred Reporting Items for Systematic reviews and Meta-Analyses
UNICEF: United Nations Children’s Fund
WHO: World Health Organization
