# Supplementary material for: Policies and guidelines supporting the sustainability of human milk donation to milk banks in Switzerland: a document analysis
Source: Int J Equity Health. 2025 Jul 31;24:214. doi: 10.1186/s12939-025-02591-3 (PMC12312558; doi:10.1186/s12939-025-02591-3)
Supplement: Supplementary file 1 — Supplementary Material 1: S1a and S1b search strategy info S2 List of Swiss human milk banks, S3 Extraction grid details, S4 PRISMA, S5 PRISMA Abstracts checklist [file 12939_2025_2591_MOESM1_ESM.docx]

Supplementary Material

Supplementary Table S1a and S1b: Search Strategy:

Table S1a: Medline (PubMed) Database (last search performed on 30/06/2024)

| Search steps | Search terms/descriptors | Number of hits |
| --- | --- | --- |
| # 1 | **(("Milk, Human"[Mesh]) OR ("Milk"[Mesh])) AND (Donor[Title/Abstract] OR Donors[Title/Abstract] OR (Donat*[Title/Abstract]) OR ("milk donation*"[Title/Abstract]) OR ("donor human milk"[Title/Abstract]) OR ("human donor milk"[Title/Abstract]) OR ("human milk donor*"[Title/Abstract]))** | 1,730 |
| # 2 | (“Guidelines as Topic”[Mesh]) OR (“Practice guidelines as Topic”[Mesh]) OR (“Guideline”[Publication Type]) OR (“Health Planning Guidelines”[Mesh]) OR (“Practice Guideline”[Publication Type]) OR (“Standard of Care”[Mesh]) OR (“Evidence-Based Practice“[Mesh]) OR (“Evidence-Based Medicine“[Mesh]) OR (“Policy“[Mesh]) OR (“Organizational Policy“[Mesh]) OR (“Public Policy“[Mesh]) OR (“Policy Making“[Mesh]) OR (“Health Policy“[Mesh]) OR Guideline*[Title/Abstract] OR recommendation*[Title/Abstract] OR Principle [Title/Abstract] OR Rule*[Title/Abstract] OR regulation*[Title/Abstract] OR polic*[Title/Abstract] OR advice*[Title/Abstract] OR Code*[Title/Abstract] OR (“proposed action*“[Title/Abstract]) OR system*[Title/Abstract] OR Procedure*[Title/Abstract] OR politic*[Title/Abstract] | 8,419,250 |
| # 3 | "Milk Banks"[Mesh] OR "milk bank*"[Title/Abstract] OR **"milkbank*"[Title/Abstract]** | 1,175 |
| # 4 | (#1 OR #3) AND #2 | 638 |

Table S1b: CINAHL Database (last search performed on 30/06/2024)

| Search steps | Search terms/descriptors | Number of hits |
| --- | --- | --- |
| # 1 | TI (milk donation*) OR AB (milk donation*) OR (MH "Donor Milk") OR TI "human donor milk" OR AB "human donor milk" OR TI "donor human milk" OR AB "donor human milk" OR TI "human milk donor*" OR AB "human milk donor*" OR ((TI Donor OR TI Donors OR TI (Donat*) OR AB Donor OR AB Donors OR AB (Donat*)) AND (AB milk OR TI milk)) | 1,055 |
| #2 | ( MH “practice guidelines” OR MH “health policy” OR MH “public policy” OR MH “policy making” OR MH “organisational policies” OR MH “politics” OR MH “Hospital policies” OR TI guidelines OR AB guidelines OR TI policy OR AB policy OR TI policies OR AB policies ) OR TI ( OR Guideline* OR recommendation* OR Principle OR Rule* OR regulation* OR polic* OR advice* OR Code* OR (proposed action*) OR system* OR Procedure* OR politic* ) OR AB ( OR Guideline* OR recommendation* OR Principle OR Rule* OR regulation* OR polic* OR advice* OR Code* OR (proposed action*) OR system* OR Procedure* OR politic* ) | 1,634,605 |
| # 3 | (MH "Milk Banks") OR (TI “milk bank*”) OR (AB “milk bank*”) | 874 |
| #4 | (#1 OR #3) AND #2 | 348 |

Table S2: List of the location of the nine Swiss milk banks (by alphabetical order of cantons):

|  | Cantons | Cities |
| --- | --- | --- |
| #1 | Aarau | Aarau |
| #2 | Basel | Basel |
| #3 | Berne | Berne |
| #4 | Graubunden | Chur |
| #5 | Luzern | Luzern |
| #6 | St Gallen* | St Gallen* (Hospital: Health Ostchweiz Kantonspital) |
| #7 | St Gallen* | St Gallen* (Hospital: Ostschweizer Kinderspital) |
| #8 | Vaud | Lausanne |
| #9 | Zurich | Zurich |

*Two milk banks are located in St Gallen in different hospitals.

Table S3: Details and guidance on the use of the document analysis grid

1. CHARACTERISTICS OF THE DOCUMENT

Data extraction

- 1. Name of the data extractor (s) and analyst(s): *List the name(s) of the data extractor(s) and analyst(s).*
  2. Authors: *Write the name of the document’s authors.*
  3. Year: *Note the year the document was published or created.*
  4. Level: *Notet whether the document is at a local, national, European or global level, (as well as cantons or milk banks when appropriate).*
  5. Type of document: *Note whether it is a policy, a set of guidelines, or other document.*
  6. Title: *List the title of the document.*
  7. Number of pages: *Write the number of pages of the document.*
  8. Doi or link: *Report the document DOI if appropriate.*
  9. Language: *Note the main language(s) of the document.*
  10. Source: *Write the source of the document (e.g. journal or website).*
  11. Edition: *Report information regarding new editions or updates to the document.*
  12. Aim, purpose, objective: *Write the document’s aim, purpose, or objective.*
  13. Target audience: *Write the target document’s audience.*
  14. Synthesis: *Synthesise the document’s most important element*.
  15. Supplementary documents: *Report any supplementary documents provided related to the topic of interest, such as consent form.*
  16. Guideline sponsor and funding: *Write the name of the guidelines’ sponsor and /or mention the funding sources for the document.*
  17. References to other relevant guidelines*: Cite the reference of any relevant guidelines mentioned in the document’s reference section.*
  18. Requisite correspondence: *Note any correspondence necessary to gather further information on the document.*

Data analysis

- 1. Data analysis: *Note any data analysis (commentary or summary) undertaken involving the characteristics of the document.*

1. ELIGIBILITY OF DOCUMENT

Data extraction

- 1. Eligibility: *State “YES” OR “NO” as well as the reason for eligibility or ineligibility.*
  2. Population or problem: *Provide a brief description.*
  3. Phenomenon of interest: *Provide a brief description.*
  4. Context: *Provide a brief description.*

Data analysis

- 1. Data analysis (commentary or summary).

1. DATA ANALYSIS I: DOCUMENT QUALITY ASSESSMENT WITH AGREE II TOOL
   1. Overall score: *Provide the overall score.*
   2. Synthesis of the evaluation: *Provide a synthesis of the evaluation.*
2. DATA ANALYSIS II: CRITERIA COMING FROM LITERATURE REVIEW
   1. Sustainability description: *Explain how sustainability is defined in the document.*
   2. Use of the term sustainability: *Explain how sustainability is used in the document (e.g. implicitly and explicitly).*
   3. Mention of the three sustainability pillars if applicable: *Write which of the three sustainability pillars are mentioned in the document (e.g. social, environmental or economic).*
   4. Social pillar*: Note how the social pillar is described in the document.*
   5. Environmental pillar: *Note how the environmental pillar is described in the document.*
   6. Economic pillar: *Note how the economic pillar is described in the document.*
   7. Use of the sustainability pillars: *Note how the sustainability pillars are used in the documents (e.g. implicitly and explicitly).*
   8. Mention of “sustainability of donation” or any variable related:  *Note whether the guidelines/policies mention the terms “sustainability of donation” or any variable related (volume of donation, recurrence of donation, duration of donation, continuing [or continuation] donating milk and number of donors), and if yes, specify which ones.*
   9. Use of the “sustainability of donation” variables: *Note how the variable described just above are used in the document.*
   10. Other variables identified: *Note any other inductively identified variables.*

Thematic analysis of factors related to the sustainability of donation, organised according to the four categories described in the systematic review.

- 1. Note any factors related to the sustainability of donation in the **donation duration category**: *Write whether the guidelines or policies mention any of the following factors: *duration of donation and * start of donation, and if yes, which ones.*
  2. Use of sustainability of donation factors*: Write how the factors related to the donation duration category are used in the document (implicitly, explicitly…).*
  3. Note any factors related to sustainability of donation in the **donors' infants’ features category**: Write whether the guidelines or policies include any of the following factors: **preterm infants, *birth weight, *admission to the neonatal intensive care unit, *thrush, *baby feeding frequency, and *growth of the baby,* and, if yes, specify which ones.
  4. Use of the sustainability of donation factors*: Write how the factors related to the donors' infants features category are used in the document (implicitly, explicitly…).*
  5. Mention any factors related to the sustainability of donation in the **donors' features category**: *Write whether the guidelines or policies mention any of the following factors: *maternal age, *number of pregnancies, *education level, *profession, *excess of milk, *pumping to stimulate lactation, *previous milk donation, * self-hydration, *diet, *physical fatigue, *presence of negative emotions, *availability of time, *experience of breastfeeding simultaneously, *frequency of milk expression, *nothing interferes with milk production, *time of day for expressing milk, and *mothers routines (going out, contraceptive use, and return to work) and, if yes, specify which ones.*
  6. Use of the sustainability of donation factors*: Write how the factors related to the donors' features category are used in the document (implicitly, explicitly…).*
  7. Note any factors related to the sustainability of donation in the **milk bank and health care category**: *Write whether the guidelines and policies include any of the following factors: *milk bank support to donation, *family support to donation, *work impact and support, *distance from milk bank and *human resources and if yes, specify which ones.*
  8. Use of the sustainability of donation factors*: Write how the factors related to the milk bank and health care category are used in the document (implicitly, explicitly…).*
  9. Other identified factors: *Note any other factors identified inductively.*
  10. Missing elements: Identify anything *missing in the documents.*
  11. Extent of document support regarding sustainability of donation: *write to what extent the document supports the sustainability of donations to Swiss milk banks (at each level: micro, meso, macro)?*
  12. Overall analysis of Section 4*: Provide an overall analysis of this section.*

1. OVERALL ANALYSIS OF THE WHOLE BODY OF DOCUMENTS: COMPARATIVE ANALYSIS OF THE DOCUMENTS
   1. Alignment of the policies and guidelines between the different levels: *Note to what extent the policies and guidelines align with different documents (local, national, European, global).*
   2. Alignment of policies and guidelines: *Write how well the guidelines align with the policies.*
   3. Comparison of documents: *Write how the documents compare in content, style, format and length.*
   4. Missing element(s): *Note any missing elements.*
   5. Reference to other included documents: *Write whether the documents contain any cross references.*
   6. Areas for improvement or suggestions: *Identify areas for improvement in the guidelines or policies and provide suggestions.*
   7. Strengths: *Write the strengths of the guidelines or policies.*

Supplementary Table S4: PRISMA 2020 Checklist

| **Section and Topic** | **Item #** | **Checklist item** | **Location of reported item** |
| --- | --- | --- | --- |
| **TITLE** | | |  |
| Title | 1 | Identify the report as a systematic review. | P14 (This systematic review is referred to as a document analysis in the title and is mentioned as a systematic review in the discussion.) |
| **ABSTRACT** | | |  |
| Abstract | 2 | See the PRISMA 2020 Abstracts checklist. | See Table S5 below. |
| **INTRODUCTION** | | |  |
| Rationale | 3 | Describe the rationale for the review in the context of existing knowledge. | p. 1 & 2 |
| Objectives | 4 | Provide an explicit statement of the objective(s) or question(s) the review addresses. | p.1 & 2 |
| **METHODS** | | |  |
| Eligibility criteria | 5 | Specify the inclusion and exclusion criteria for the review and how studies were grouped for the syntheses. | p. 4 & 5 |
| Information sources | 6 | Specify all databases, registers, websites, organisations, reference lists and other sources searched or consulted to identify studies. Specify the date when each source was last searched or consulted. | p. 4 & Table S1a and S1b, Table S2 |
| Search strategy | 7 | Present the full search strategies for all databases, registers and websites, including any filters and limits used. | p. 4 & 5, Tables S1a, S1b, S2. |
| Selection process | 8 | Specify the methods used to decide whether a study met the inclusion criteria of the review, including how many reviewers screened each record and each report retrieved, whether they worked independently, and if applicable, details of automation tools used in the process. | p. 4 & 5 |
| Data collection process | 9 | Specify the methods used to collect data from reports, including how many reviewers collected data from each report, whether they worked independently, any processes for obtaining or confirming data from study investigators, and if applicable, details of automation tools used in the process. | p. 4 & 5. |
| Data items | 10a | List and define all outcomes for which data were sought. Specify whether all results that were compatible with each outcome domain in each study were sought (e.g. for all measures, time points, analyses), and if not, the methods used to decide which results to collect. | p. 5 & Table S3 |
|  | 10b | List and define all other variables for which data were sought (e.g. participant and intervention characteristics, funding sources). Describe any assumptions made about any missing or unclear information. | p. 5 & Table S3 |
| Study risk of bias assessment | 11 | Specify the methods used to assess risk of bias in the included studies, including details of the tool(s) used, how many reviewers assessed each study and whether they worked independently, and if applicable, details of automation tools used in the process. | p. 5 |
| Effect measures | 12 | Specify for each outcome the effect measure(s) (e.g. risk ratio, mean difference) used in the synthesis or presentation of results. | Not applicable |
| Synthesis methods | 13a | Describe the processes used to decide which studies were eligible for each synthesis (e.g. tabulating the study intervention characteristics and comparing against the planned groups for each synthesis (item #5)). | p. 5 |
|  | 13b | Describe any methods required to prepare the data for presentation or synthesis, such as handling of missing summary statistics, or data conversions. | p. 5 |
|  | 13c | Describe any methods used to tabulate or visually display results of individual studies and syntheses. | p. 5 |
|  | 13d | Describe any methods used to synthesize results and provide a rationale for the choice(s). If meta-analysis was performed, describe the model(s), method(s) to identify the presence and extent of statistical heterogeneity, and software package(s) used. | p. 5 |
|  | 13e | Describe any methods used to explore possible causes of heterogeneity among study results (e.g. subgroup analysis, meta-regression). | Not applicable |
|  | 13f | Describe any sensitivity analyses conducted to assess robustness of the synthesized results. | Not applicable |
| Reporting bias assessment | 14 | Describe any methods used to assess risk of bias due to missing results in a synthesis (arising from reporting biases). | Not applicable |
| Certainty assessment | 15 | Describe any methods used to assess certainty (or confidence) in the body of evidence for an outcome. | p. 5 |
| **RESULTS** | | |  |
| Study selection | 16a | Describe the results of the search and selection process, from the number of records identified in the search to the number of studies included in the review, ideally using a flow diagram. | p.6 & figure 1 |
|  | 16b | Cite studies that might appear to meet the inclusion criteria, but which were excluded, and explain why they were excluded. |  |
| Study characteristics | 17 | Cite each included study and present its characteristics. | p. 6-10, Table 1 & Table 2 |
| Risk of bias in studies | 18 | Present assessments of risk of bias for each included study. |  |
| Results of individual studies | 19 | For all outcomes, present, for each study: (a) summary statistics for each group (where appropriate) and (b) an effect estimate and its precision (e.g. confidence/credible interval), ideally using structured tables or plots. | Not applicable |
| Results of syntheses | 20a | For each synthesis, briefly summarise the characteristics and risk of bias among contributing studies. | Table 1 and table 2 |
|  | 20b | Present results of all statistical syntheses conducted. If meta-analysis was done, present for each the summary estimate and its precision (e.g. confidence/credible interval) and measures of statistical heterogeneity. If comparing groups, describe the direction of the effect. | p. 6-13 and Table 3 |
|  | 20c | Present results of all investigations of possible causes of heterogeneity among study results. |  |
|  | 20d | Present results of all sensitivity analyses conducted to assess the robustness of the synthesized results. |  |
| Reporting biases | 21 | Present assessments of risk of bias due to missing results (arising from reporting biases) for each synthesis assessed. |  |
| Certainty of evidence | 22 | Present assessments of certainty (or confidence) in the body of evidence for each outcome assessed. |  |
| **DISCUSSION** | | |  |
| Discussion | 23a | Provide a general interpretation of the results in the context of other evidence. | p. 14-17 |
|  | 23b | Discuss any limitations of the evidence included in the review. | p. 14-17 |
|  | 23c | Discuss any limitations of the review processes used. | p. 17 |
|  | 23d | Discuss implications of the results for practice, policy, and future research. | p. 14-17 |
| **OTHER INFORMATION** | | |  |
| Registration and protocol | 24a | Provide registration information for the review, including register name and registration number, or state that the review was not registered. |  |
|  | 24b | Indicate where the review protocol can be accessed, or state that a protocol was not prepared. | p. 4 |
|  | 24c | Describe and explain any amendments to information provided at registration or in the protocol. |  |
| Support | 25 | Describe sources of financial or non-financial support for the review, and the role of the funders or sponsors in the review. |  |
| Competing interests | 26 | Declare any competing interests of review authors. | p. 18 |
| Availability of data, code and other materials | 27 | Report which of the following are publicly available and where they can be found: template data collection forms; data extracted from included studies; data used for all analyses; analytic code; any other materials used in the review. | Template data collection form (word document, not the Excel version) |

*From:*  Page MJ, McKenzie JE, Bossuyt PM, Boutron I, Hoffmann TC, Mulrow CD, et al. The PRISMA 2020 statement: An updated guideline for reporting systematic reviews. BMJ 2021;372:n71. doi: 10.11

Supplementary Table S5: PRISMA 2020 Abstracts Checklist

| **Section and Topic** | **Item #** | **Checklist item** | **Reported (Yes/No)** |
| --- | --- | --- | --- |
| **TITLE** | | |  |
| Title | 1 | Identify the report as a systematic review. | Systematic review referred to as a document analysis. |
| **BACKGROUND** | | |  |
| Objectives | 2 | Provide an explicit statement of the main objective(s) or question(s) the review addresses. | Yes |
| **METHODS** | | |  |
| Eligibility criteria | 3 | Specify the inclusion and exclusion criteria for the review. | Yes, some briefly. |
| Information sources | 4 | Specify the information sources (e.g. databases, registers) used to identify studies and the date when each was last searched. | Yes |
| Risk of bias | 5 | Specify the methods used to assess risk of bias in the included studies. | No |
| Synthesis of results | 6 | Specify the methods used to present and synthesise results. | Yes |
| **RESULTS** | | |  |
| Included studies | 7 | Give the total number of included studies and participants and summarise relevant characteristics of studies. | Yes |
| Synthesis of results | 8 | Present results for main outcomes, preferably indicating the number of included studies and participants for each. If meta-analysis was done, report the summary estimate and confidence/credible interval. If comparing groups, indicate the direction of the effect (i.e. which group is favoured). | Yes |
| **DISCUSSION** | | |  |
| Limitations of evidence | 9 | Provide a brief summary of the limitations of the evidence included in the review (e.g. study risk of bias, inconsistency and imprecision). | No |
| Interpretation | 10 | Provide a general interpretation of the results and important implications. | Yes |
| **OTHER** | | |  |
| Funding | 11 | Specify the primary source of funding for the review. | No |
| Registration | 12 | Provide the register name and registration number. | No |

*From:*  Page MJ, McKenzie JE, Bossuyt PM, Boutron I, Hoffmann TC, Mulrow CD, et al. The PRISMA 2020 statement: an updated guideline for reporting systematic reviews. BMJ 2021;372:n71. doi: 10.1136/bmj.n71
